# Genetic Evidence Supporting a Causal Association Between mTORC1-Dependent Circulating Protein Levels and Diabetic Retinopathy

**DOI:** 10.1167/tvst.14.5.4

**Published:** 2025-05-02

**Authors:** Yaqi Bai, Yujia Xi, Chenwei Gui, Guohai Huang, Guohong Zhou

**Affiliations:** 1 The First Clinical Medical College, Shanxi Medical University, Taiyuan, Shanxi, China; 2 The Second Hospital of Shanxi Medical University, Department of Urology, Taiyuan, China; 3 Department of Ophthalmology, Shanxi Eye Hospital Affiliated to Shanxi Medical University, Taiyuan, Shanxi, China

**Keywords:** mTORC1-dependent protein, diabetic retinopathy, Mendelian randomization, EIF4E, causal association

## Abstract

**Purpose:**

The mechanistic target of rapamycin (mTOR) signaling pathway is essential for the onset and progression of diabetic retinopathy (DR). Nevertheless, the impact of mTORC1 downstream proteins in DR remains uncertain. Therefore, we performed a Mendelian randomization (MR) research to assess the causal effect of downstream mTORC1 proteins on DR risk.

**Methods:**

Summary statistics on mTORC1 downstream proteins and DR were obtained from the INTERVAL and FinnGen studies (14,584 patients and 176,010 controls), respectively. We used various MR techniques, including inverse-variance-weighted, weighted median, and MR-Egger. Possible pleiotropy and heterogeneity were identified through sensitivity analysis.

**Results:**

Genetically predicted eIF4E was positively correlated to DR risk (odds ratio = 1.057; 95% confidence interval, 1.008–1.109; *P* = 0.022]. No relationship has been shown for circulating RP-S6K, eIF4G, eIF4A, eIF4E-BP and eIF4B levels with DR formation. There was no heterogeneity or unbalanced level pleiotropy identified.

**Conclusions:**

Higher levels of serum eIF4E promote the progression of DR, proposing that pharmacological inhibition of eIF4E activity may be a prospective DR therapeutic strategy.

**Translational Relevance:**

The present study has highlighted the role of eIF4E in the development of DR, establishing the foundation for basic research into DR targets.

## Introduction

As the morbidity of diabetes rises worldwide, diabetic retinopathy (DR), its most universal and severe microvascular complication, remains the leading cause of moderate to severe vision loss and blindness worldwide.^1^^,^^2^ In DR, chronic hyperglycemia alters the retinal structure and abducts changes in neurons and vascular cells through multiple mechanisms, including inflammation, neurodegeneration, oxidative stress, and angiogenesis, leading to visual impairment and blindness.^1^ Despite advances in treatment, current therapeutic strategies have yet to achieve significant clinical improvements in vision for patients.^3^ The global incidence and impact of DR are anticipated to skyrocket in the following decades, rising from approximately 103 million instances in 2020 to 130 million in 2030 and by extension to 161 million by 2045.^4^ To address the growing threat, exploring more effective targets plays a role in guiding clinical practice for the prevention and treatment of DR.

Some evidence suggests the mechanistic target of the rapamycin (mTOR) signaling channel has an important effect on ocular disorders.^5^ In mammals, serine/threonine protein kinases are considered targets of mTOR, which bind to multiple proteins, forming two different complexes, mTORC1 and mTORC2.^6^ Compared to mTORC2, mTORC1 is more sensitive to rapamycin and generally engaged in macromolecule synthesis, cell proliferation, inflammatory mediator production, and autophagy.^7^ mTORC1 promotes cap-dependent protein synthesis via phosphorylating eukaryotic initiation factor 4E binding protein (eIF4E-BP) and ribosomal protein S6 kinase (RP-S6K). eIF4E-BP is a repressor of the eukaryotic translation initiation complex eIF4F (eIF4E, eIF4G, and eIF4A), and its phosphorylation dissociates itself from eIF4E, facilitating the synthesis of eIF4F and beginning of 5′cap-dependent translation.^8^ Conversely, RP-S6K phosphorylates eIF4B, which positively modulates the eIF4F complex.^9^

Immunolocalization of rat, mouse, and human retinas discovered the high expression of mTORC pathways in the medial retina with active mTORC1 signaling.^10^ Many studies have demonstrated that direct mTOR inhibition participated in controlling angiogenesis, inflammatory progression, and anti-apoptosis.^11^^,^^12^ However, enhanced mTOR signaling can reduce retinal cell apoptosis in a rat model of DR,^13^ seemingly contradicting the potential benefits of mTOR suppression in DR. Hence, it is necessary to confirm the absence or existence of the causal association between mTORC1-associated circulating proteins and DR and to determine whether such a relationship is protective or detrimental.

Mendelian randomization (MR), an analytical method using genome-wide association studies (GWAS), uses genetic variants as instrumental variables (IVs) to assess the causal relationship of exposure on outcome.^14^ This approach minimizes confounding factors and addresses the issue of reverse causality.^15^ Applying summary GWAS statistics from European populations, we conducted a two-sample MR research for the estimation of the connection between downstream mTORC1 protein levels(eIF4E-BP, eIF4A, eIF4G, eIF4E, RP-S6K and eIF4B) and DR.

## Material and Methods

### Study Design

**Figure 1.**
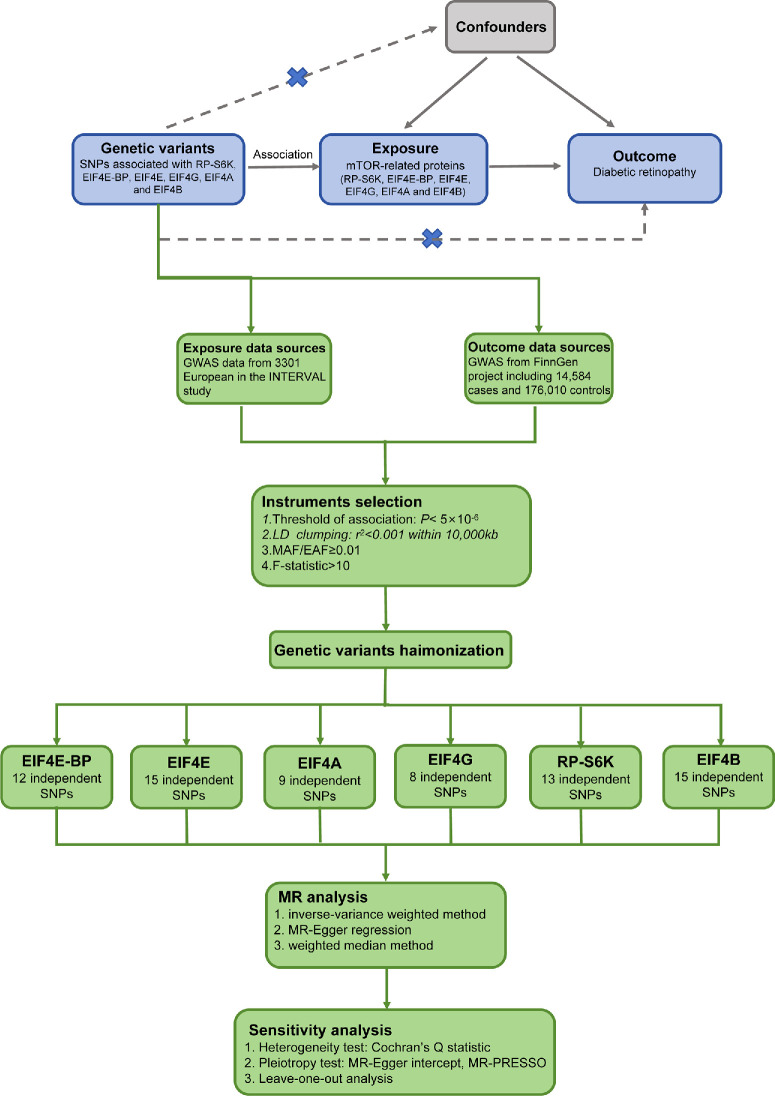
The flow chart of the Mendelian randomization study.

The MR analysis is conducted conforming the STROBE-MR guidelines based on GWAS data.^14^ MR analysis relies on three core assumptions.^16^ First, genetic variation should be strongly connected with downstream mTORC1 proteins. Second, IV must be independent of any confounding variables. Finally, IV impacts DR only through mTOR proteins. The flow chart is displayed in Figure 1.

### Data Source

Genetic variation in mTORC1-dependent proteins were derived through the publicly accessible Human Plasma Proteome Atlas, which acquired 3622 plasma proteins from 3301 volunteers in the INTERVAL trial.^17^ Between mid-2012 and mid-2014, the INTERVAL research is a randomized experiment evaluating the frequency of blood donation on a genomic bioresource of approximately 50,000 volunteers over 18 years old enrolled from England's National Health Service Blood and Transplant centers.^18^ These 3622 plasma proteins were quantified through a multiplexed, aptamer-based approach (SOMAscan assay), which widens inferior limit of the detected protein abundance. The results identified 1927 genotype-protein associations (pQTLs), 89% of which had never been reported before, indicating prospective treatment targets.

Summary-level data for DR was derived from the FinnGen study, which investigated 500,000 Finns, involving a GWAS for 1932 clinical endpoints and enriching data on rare and late-onset disorders. The GWAS contained 14,584 DR cases and 176,010 controls. For analysis, age, gender, genotyping batches, genetic correlation, and the top 10 major components were modulated. A detailed overview of all data sources can be found in Table 1.

**Table 1.** All Detailed Data Sources

Trait	Consortium	Sample Size	Population	Dataset
eIF4E	NA	3301	European	Prot-a-923
eIF4G	NA	3301	European	Prot-a-925
eIF4A	NA	3301	European	Prot-a-921
eIF4E-BP	NA	3301	European	Prot-a-924
RP-S6K	NA	3301	European	Prot-a-2596
eIF4B	NA	3301	European	Prot-a-922
DR	NA	14,584	European	Finn-b-DM_RETINOPATHY_EXMORE

### Instrument Variable Selection

Because only two to five SNPs were obtained using a threshold of *P* < 5 × 10^−^^8^, with few effective IVs, we chose a *P* < 5 × 10^−^^6^ and *r*^2^ < 0.001 threshold to collect enough and independent SNPs and evaluate precise results.^19^^,^^20^ SNPs with palindromic sequences and effect allele frequencies(EAF) < 0.01 were eliminated. For these IVs, we computed the *F* statistics as follows: *F* = R^2^ (N − K − 1)/[K(1 − R^2^)]. SNPs with *F* < 10 were removed to prevent weak instrumental bias. SNPs significantly related to the outcome were excluded (*P* > 1.0 × 10^−^^5^) to address potential confounding effects. In addition, we used the PhenoScanner V2 database to confirm whether IV was related to other traits and excluded any relevant indicators of potential confounders of DR, including smoking, glucose level, glycosylated hemoglobin (HbA1c), diabetes, hypertension, obesity, dyslipidemia, puberty and pregnancy.^1^

### Statistical Analysis

Three techniques, including inverse variance weighted (IVW), weighted median estimator (WM) and MR-Egger were applied to perform MR study. The primary approach was IVW model, which utilized a weighted linear regression model to assess the causality of each SNP on the result, and meta-analysis that pooled the results of individual SNP to determine the overall causal effect.^21^ The WM^22^ and MR-Egger^23^ methods complement the IVW methods by offering more trustworthy calculations on a larger scale but they are less efficient. And the odds ratio (OR) value was used to assess the possible causal association between mTORC1-dependent proteins and DR.

### Sensitivity Analysis

We determined horizontal pleiotropy through the MR-Egger interception method. If it had a *P* value < 0.05, the IVW estimate might be biased.^24^ And the funnel plot was applied to depict directional pleiotropy. The scatter plot demonstrated the link between mTORC1 proteins and DR. Meanwhile, the horizontal pleiotropy was corrected through MR Pleiotropy Residual Sum and Outlier (MR-PRESSO) test, which discovered and removed abnormal SNPs, then recalculate causality.^25^ Furthermore, Cochran's Q test evaluated potential heterogeneity among IVs, with *P* values < 0.05 representing the presence of heterogeneity.^26^ To ensure the reliability of the outcomes, leave-one-out analysis identified whether any single SNP tainted effect calculations.^15^ The “mr.raps,” “MR-PRESSO,” and “TwoSampleMR” packages in R software, Version 4.1.2 were used for statistical analyses.

## Results

### Instrumental Variables for MR

Our screening procedure was strictly based on three IV assumptions. After an initial stage of quality control steps, 15,8,9,12, 13, and 15 SNPs in DR were identified as IVs related to eIF4E, eIF4G, eIF4A, eIF4E-BP, RP-S6K, and eIF4B, respectively. The *F* statistic for each SNP ranged between 21.23 and 362.95, indicating adequate validity of selected SNPs. Details of the identified SNPs relating to DR are presented in Supplementary Table 1.

### MR Analysis Results

**Figure 2.**
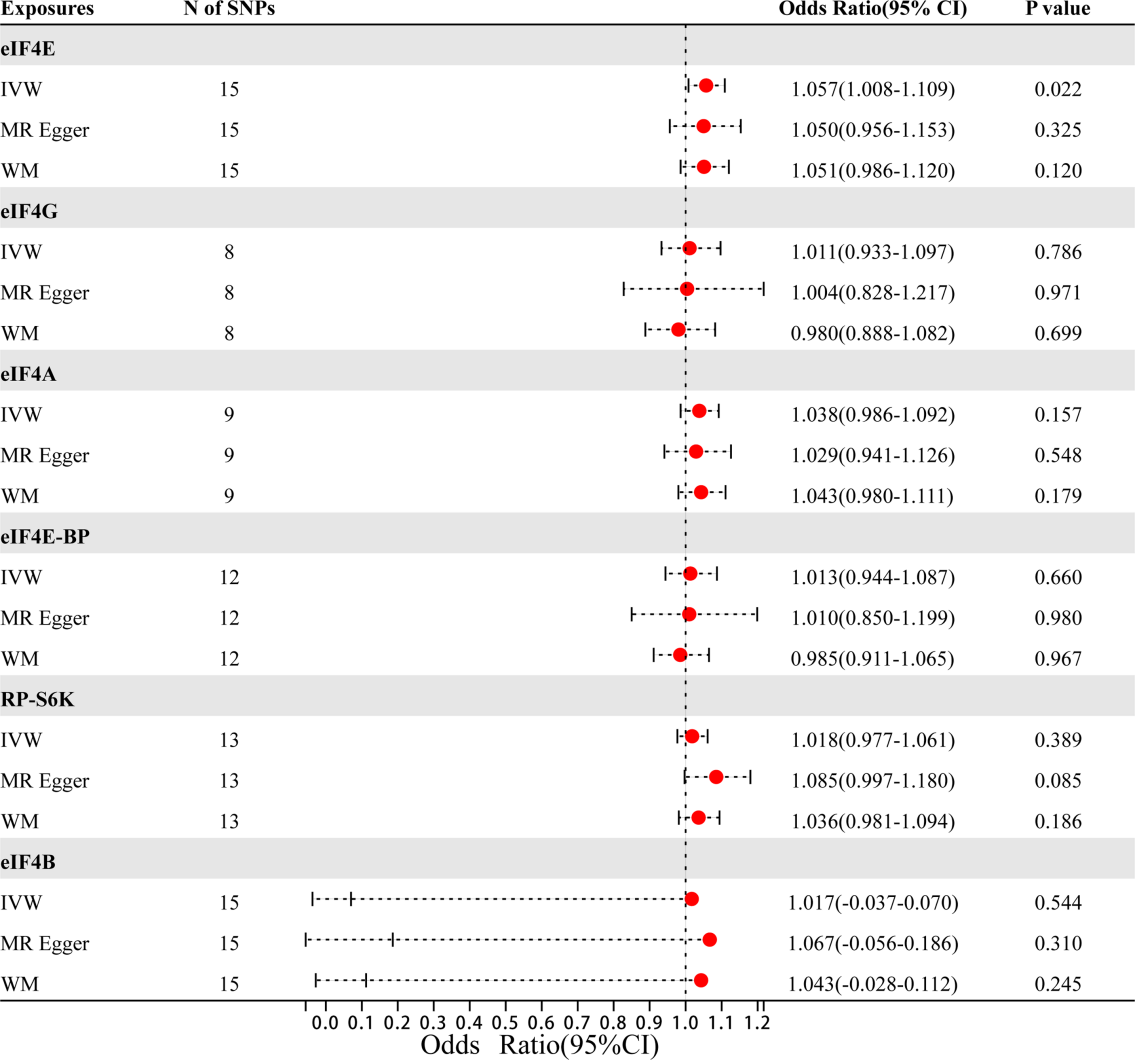
MR analysis estimates of mTORC1-related proteins and the risk of DR.

**Figure 3.**
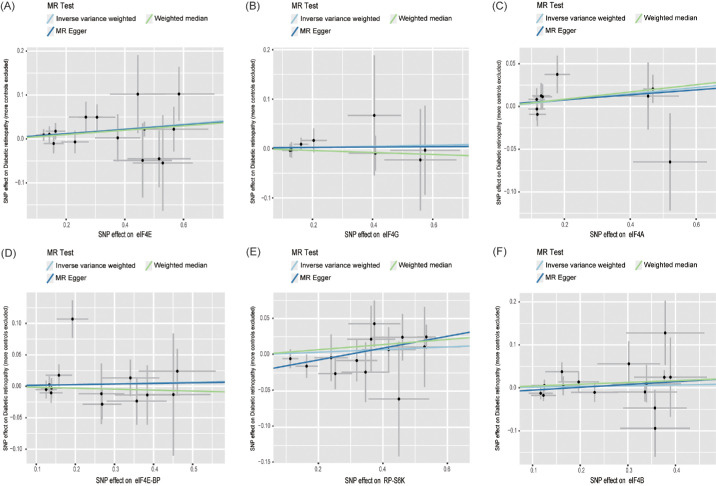
Scatter plot of the causal relationship between mTORC1-related proteins and DR. (**A**) eIF4E; (**B**) eIF4G; (**C**) eIF4A; (**D**) eIF4E-BP; (**E**) RP-S6K; (**F**) eIF4B.

The exact findings of this MR study are given in Figure 2. The IVW technique revealed that plasma eIF4E protein levels (OR = 1.057; 95% confidence [CI], 1.008–1.109; *P* = 0.022) were statistically related to an elevated DR risk. The scatter plot (Fig. 3) indicates that the same causality was demonstrated using the other two approaches. Nevertheless, analysis of the effects of RP-S6K (OR = 1.018; 95% CI, 0.977–1.061; *P* = 0.389), eIF4A (OR = 1.038; 95% CI, 0.986–1.092; *P* = 0.157), eIF4G (OR = 1.011; 95% CI, 0.933–1.097; *P* = 0.786), eIF4E-BP (OR = 1.013; 95% CI, 0.944–1.087; *P* = 0.660), and eIF4B (OR = 1.017; 95% CI, 0.037–1.070; *P* = 0.544) levels on DR risk were not statistically meaningful.

### Sensitivity Analysis Results

**Figure 4.**
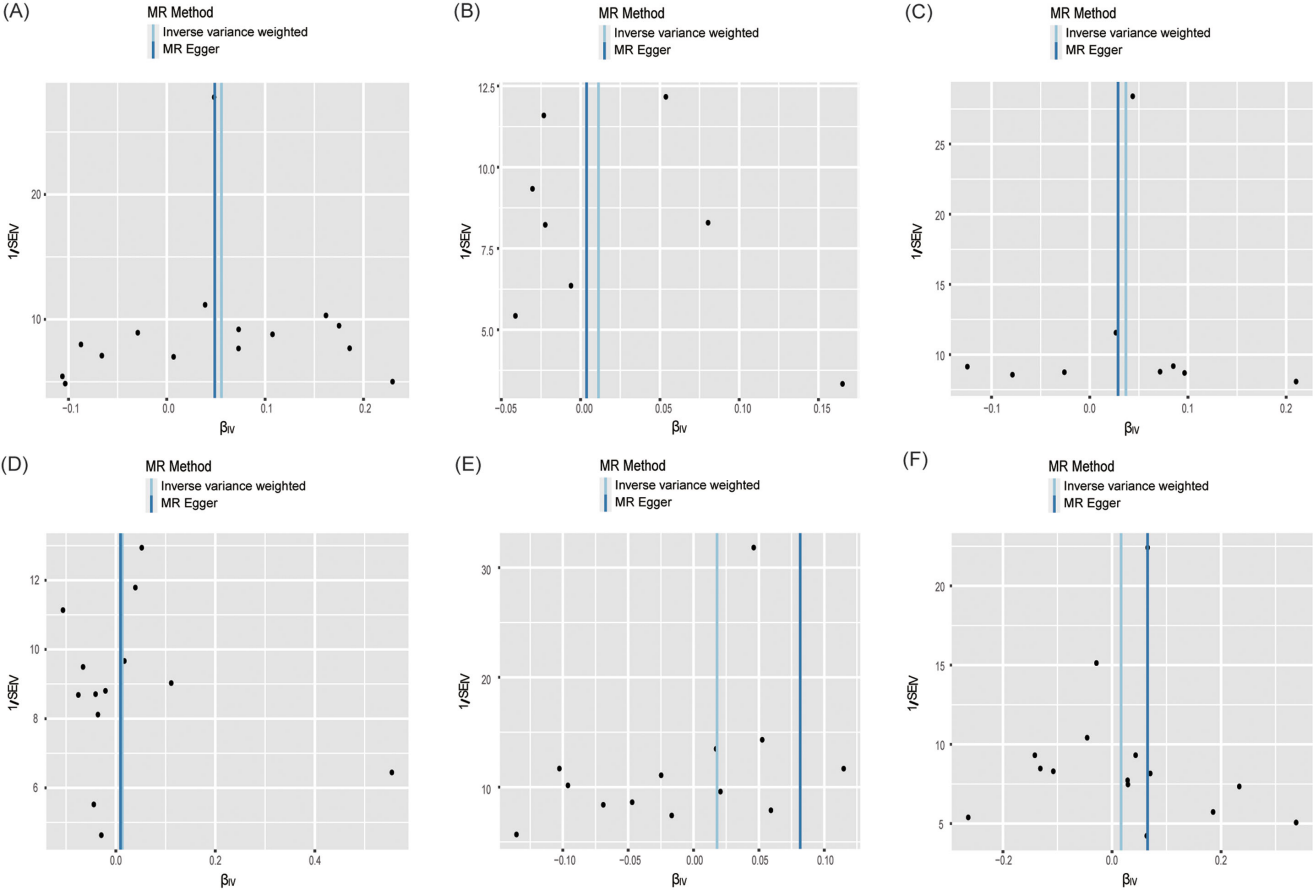
Funnel plot of the causal relationship mTORC1 related proteins and DR. (**A**) eIF4E; (**B**) eIF4G; (**C**) eIF4A; (**D**) eIF4E-BP; (**E**) RP-S6K; (**F**) eIF4B.

**Figure 5.**
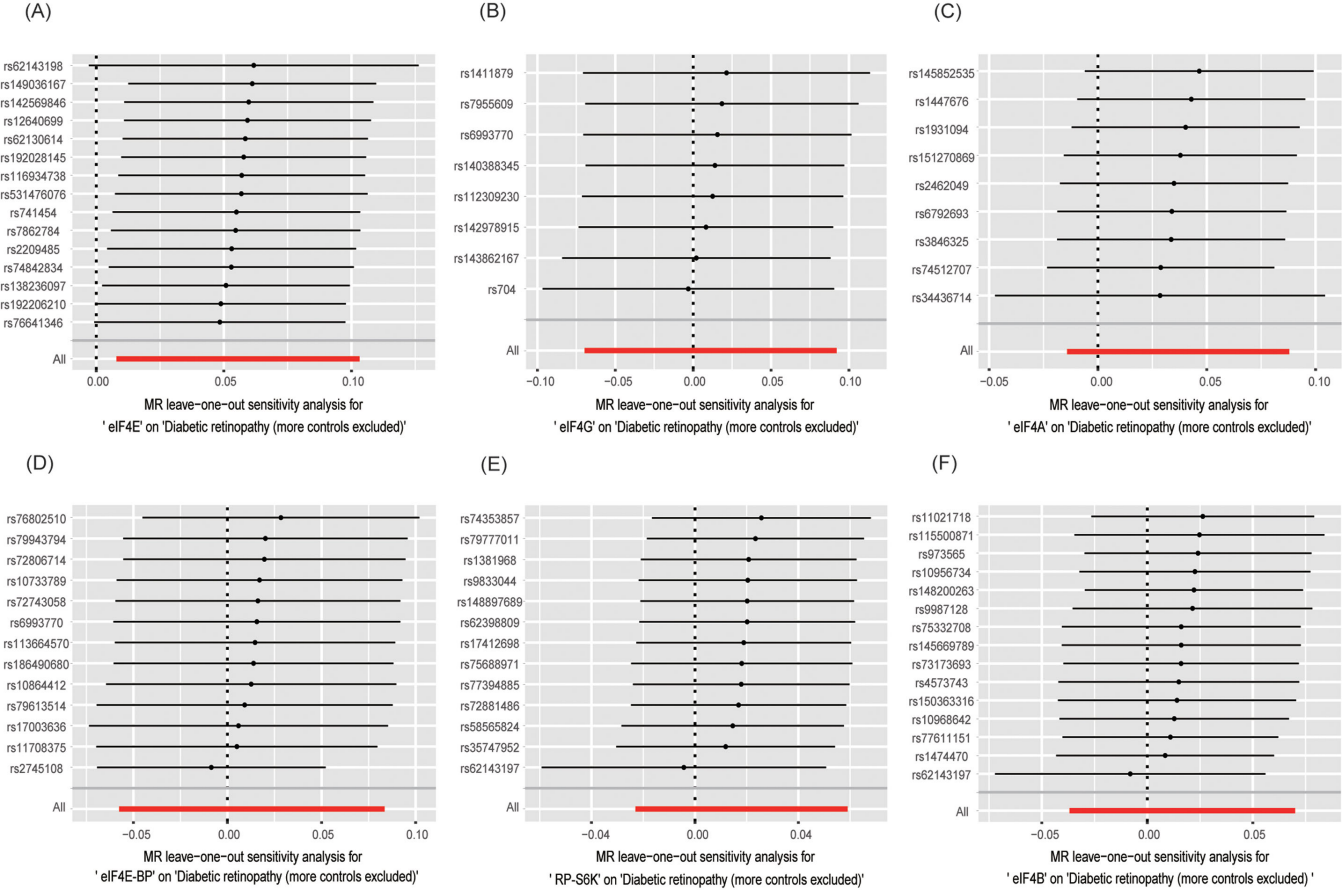
Leave-one-out analysis for the estimates of mTORC1 related proteins on DR. (**A**) eIF4E; (**B**) eIF4G; (**C**) eIF4A; (**D**) eIF4E-BP; (**E**) RP-S6K; (**F**) eIF4B.

Cochran's Q test found no heterogeneity (all *P* > 0.05). Moreover, all *P* values for MR-Egger regression intercepts were greater than 0.05, with no unbalanced pleiotropy (Table 2), which was validated through the almost symmetrical funnel plot (Fig. 4). Meanwhile, MR-PRESSO could not find outliers in any of the exposures, indicating the absence of pleiotropic bias. Finally, the leave-one-out analysis discovered that none of the SNPs significantly affected the findings of this MR study, further confirming the data robustness (Fig. 5).

**Table 2.** Sensitivity Analysis for the Relationship of mTORC1-Related Proteins on DR

Exposure	Cochran's Q	Cochran's Q *P*-Value	MR-Egger Intercept	MR-Egger Intercept *P*-Value	MR-PRESSO *P*-Value
eIF4E	8.685	0.851	0.002	0.873	0.880
eIF4G	1.340	0.987	0.001	0.936	0.990
eIF4A	6.024	0.645	0.002	0.839	0.714
eIF4E-BP	16.847	0.120	0.001	0.826	0.216
RP-S6K	7.645	0.812	−0.023	0.119	0.753
eIF4B	15.594	0.339	−0.012	0.395	0.553

## Discussion

This is the first MR research using GWAS data for the evaluation of causal effect between mTORC1 protein levels and DR. The findings revealed a positive direct relationship between plasma eIF4E protein levels and DR. However, no specific evidence was discovered supporting a connection between genetically determined RP-S6K, eIF4A, eIF4G, eIF4E-BP, eIF4B, and DR risk. The absence of significant pleiotropy and heterogeneity in our MR analysis enhances the credibility of our causal inference. These results suggest that the observed association between mTORC1 signaling and DR is biologically plausible and unlikely to be confounded by alternative pathways or biased by inconsistent genetic effects.

There has been evidence that mTOR modulates DR by mediating oxidative stress, autophagy and cell death.^27^ However, the involvement of mTORC1 pathway downstream proteins has not been demonstrated. The retina, being a highly metabolically active tissue, demands a substantial supply of oxygen and nutrients. Under hyperglycemic conditions, retinal vessels exhibit impaired oxygen delivery, and retinal tissues experience reduced oxygen use, exacerbating the pathological progression of DR.^28^ During DR, hypoxia triggers the transcriptional production of vascular endothelial growth factor (VEGF) in the retina, which promotes vascular endothelial cell proliferation, increases vascular permeability, and drives pathological neovascularization.^29^ The regulation of VEGF expression is closely associated with the structural features of its mRNA, particularly the 5′-untranslated region (5′-UTR), which significantly influences translation efficiency. Cap-dependent translation of VEGF mRNA initiates when the 40S ribosomal subunit binds to the m7GTP cap structure within the 5′-UTR, a process mediated by the eIF4F complex. As the core subunit of eIF4F, eIF4E recognizes and binds to the 5′-end cap of mRNA, playing an essential role in cap-dependent translation initiation.^30^ Furthermore, the expression of VEGF, eIF4E, and eIF4E-BP were elevated in the retinas of diabetic rats.^31^ Hence, hyperglycemia activates the mTORC1 signaling pathway, leading to the phosphorylation of 4E-BP1 and subsequent release of eIF4E. The eIF4E recognizes the 5′-end cap structure of VEGF mRNA, facilitating its translation and enhancing VEGF expression.^32^

DR is now generally regarded as a neurodegenerative disease characterized by reactive gliosis and apoptosis of retinal neurons, which is closely connected with the adjustment of autophagy.^33^ Autophagy is a catabolic phenomenon that eliminate, digest, and recycle organelles and proteins.^34^ In DR, hyperglycemia-induced oxidative stress and endoplasmic reticulum stress can lead to autophagy failure, causing damage to retinal vascular endothelial cells and pericytes.^33^ Rapamycin (mTOR inhibitor) recovers the autophagic mechanism and inhibits apoptosis in glial cells, thus suggesting that dysregulation of autophagy can be a significant target for preventing DR deterioration.^35^ The mTORC1 signaling pathway serves as a critical negative regulator of autophagy. By phosphorylating ULK1 (Unc-51-like kinase 1), a key kinase responsible for autophagy initiation, mTORC1 inhibits the activation of ULK1 and disrupts its interaction with AMP-activated protein kinase (AMPK), ultimately suppressing autophagy initiation.^36^ Bioinformatics analysis reveals eIF4E as a central component of the autophagy pathway.^37^ Protein imprinting analysis revealed that phosphorylation of 4E-BP1 and subsequent release of eIF4E downregulated the expression of pro-apoptotic protein Bax and autophagy-related proteins, including Beclin-1, LC3B-II, and ATG5. Conversely, the levels of anti-apoptotic protein Bcl-2 were upregulated.^38^ Additionally, phosphorylated mTORC1 was decreased in diabetic retinas, accompanied with upregulation of Beclin-1 and LC3.^39^ Therefore we hypothesize that eIF4E suppresses autophagy and exacerbates retinal cell damage by enhancing the translation of anti-apoptotic proteins, while concurrently downregulating the expression of autophagy-related proteins. However, this hypothesis needs to be verified in the future.

So far, researches indicated the great function of various drugs acting on mTOR in DR treatment. REDD1 inhibits 4E-BP1 phosphorylation and eIF4E release by suppressing the Akt/mTORC1 signaling pathway in diabetic rodent retinas.^40^ Mingmu Xiaomeng pills could protect the diabetic retina by decreasing the phosphorylation of mTORC1 and associated proteins in rats.^41^ Similarly, berberine ameliorates DR by completely inhibiting HIF-1α/VEGF expression through Akt/mTOR pathways.^42^ In addition, INK128 selectively inhibited downstream effector 4E-BP1 and p70 S6 kinase 1, preventing the RPE cells migration, whereas other mTORC1 targets were virtually impervious.^43^ Their findings suggested that this selective inhibition of mTORC1 may be a hopeful treatment for DR. Our research discovered a clear causal relationship of eIF4E with DR. Hence, we assumed that selective regulation of mTORC1 targets, such as eIF4E, mitigates DR, and this method should be explored more in the future to exploit specific therapies for DR.

Our study carried out MR analysis which uses genetic variation as IVs. Genetic variation is irrelevant to lifestyle, social background, and other characteristics, avoiding the influence of confounders and eliminating reverse causality. Second, we used multiple MR techniques, including IVW, MR-Egger, and WM to robustly assess causality. Then MR-Egger and MR-PRESSO methods confirmed that pleiotropy was almost nonexistent. Third, there was no overlap between exposure and results in our study, leading to strong statistical efficacy for most analyses. This research has certain limitations. First, patients in the current study are all of European descent, limiting the generalizability of our findings to other populations, and further replication and validation of this discovery in other ethnic groups is required. Second, although the impacts of pleiotropy and heterogeneity were not uncovered by the sensitivity analysis, there may be underlying heterogeneity and partial pleiotropy resulting from existing differences in the contained population. Third, because the study relied on aggregate statistics rather than individual-level data, subgroup analyses were not possible. Finally, The absence of SNP-specific sample sizes (n-values) in the dataset ( finn-b-DM_RETINOPATHY_EXMORE) precluded colocalization and summary-data-based Mendelian randomization analyses, which introduces potential residual confounding. Although our sensitivity analysis demonstrated robustness against horizontal pleiotropy, these approaches cannot fully substitute for colocalization evidence. To substantiate our preliminary causal inferences between mTOR downstream proteins and DR, future investigations will prioritize more complete GWAS datasets containing detailed SNP-level metadata (standard errors, allele frequencies, and n-values) or leverage collaborative access to individual-level phenotypic-genotypic data.

## Conclusions

In summary, our unbiased two-sample MR research confirms a causal association between circulating EIF4E levels and an elevated DR risk. However, there was no causal relationship between RP-S6K, eIF4G, eIF4A, eIF4E-BP and eIF4B and DR. Further high-quality studies are needed for the exact functions and long-term role of EIF4E on DR. Nevertheless, the results of this research build the groundwork for more valid pharmacological targets for DR prevention and therapy.

## Supplementary Material

Supplement 1
